# Supplementary material for: The effect of foot-stretcher position and stroke rate on ergometer rowing kinematics
Source: PLoS One. 2023 May 11;18(5):e0285676. doi: 10.1371/journal.pone.0285676 (PMC10174490; doi:10.1371/journal.pone.0285676)
Supplement: S1 File — (PPTX) [file pone.0285676.s001.pptx]

## Slide 1
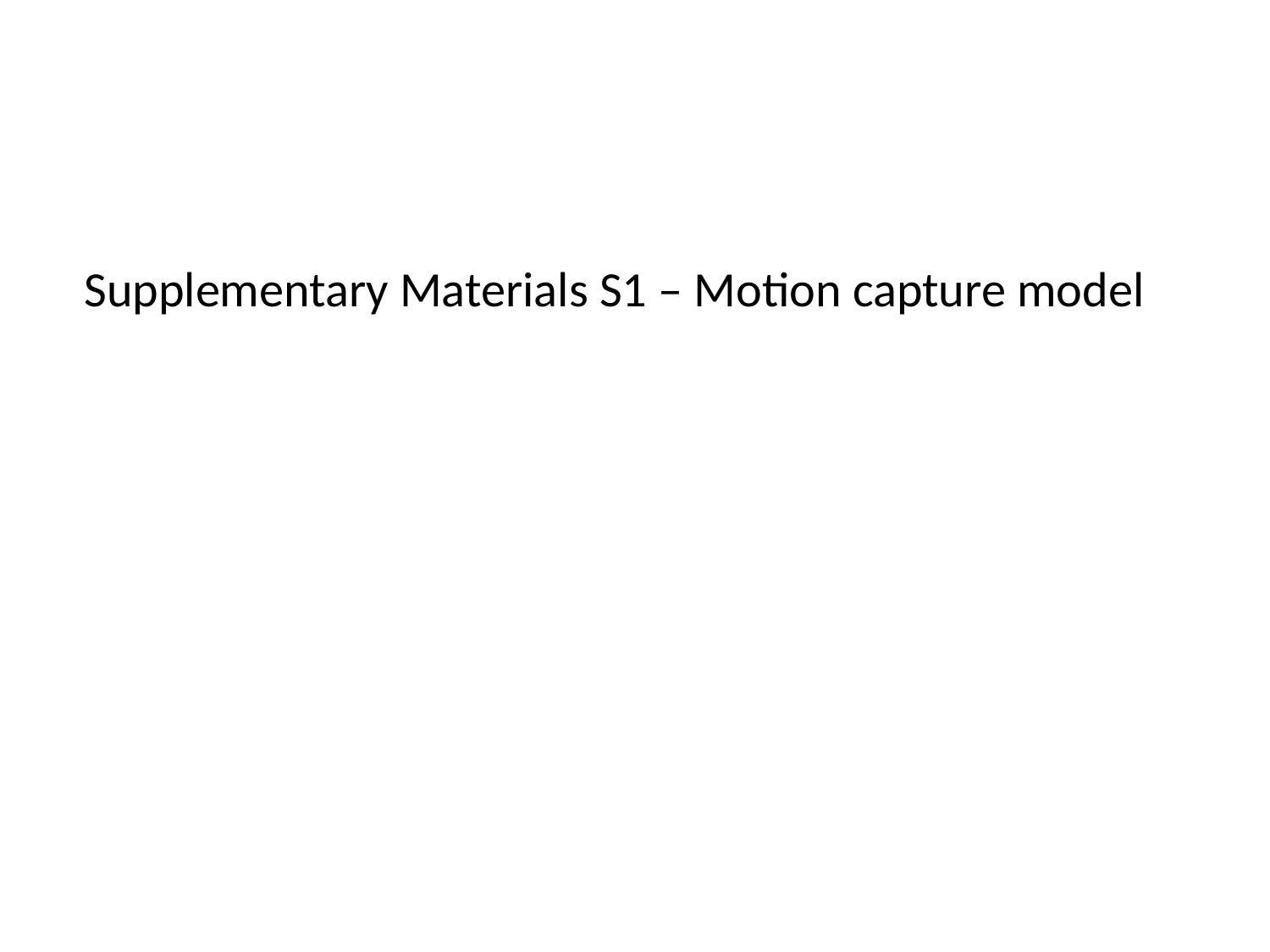

Supplementary Materials S1 – Motion capture model

## Slide 2
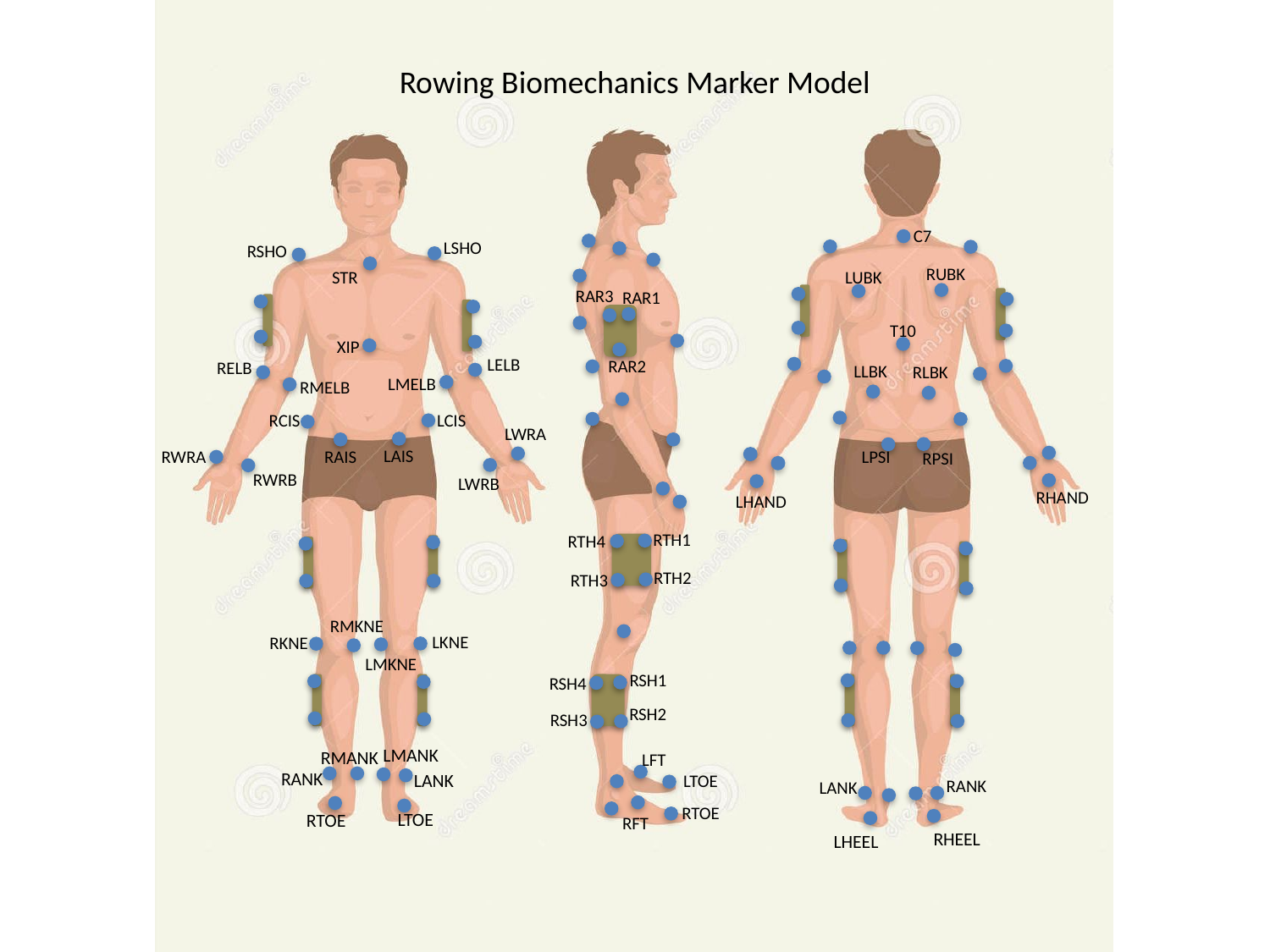

Rowing Biomechanics Marker Model
C7
LSHO
RSHO
RUBK
STR
LUBK
RAR3
RAR1
T10
XIP
LELB
RAR2
RELB
LLBK
RLBK
LMELB
RMELB
RCIS
LCIS
LWRA
LAIS
RWRA
RAIS
LPSI
RPSI
RWRB
LWRB
RHAND
LHAND
RTH1
RTH4
RTH2
RTH3
RMKNE
LKNE
RKNE
LMKNE
RSH1
RSH4
RSH2
RSH3
LMANK
RMANK
LFT
RANK
LANK
LTOE
RANK
LANK
RTOE
LTOE
RTOE
RFT
RHEEL
LHEEL

## Slide 3
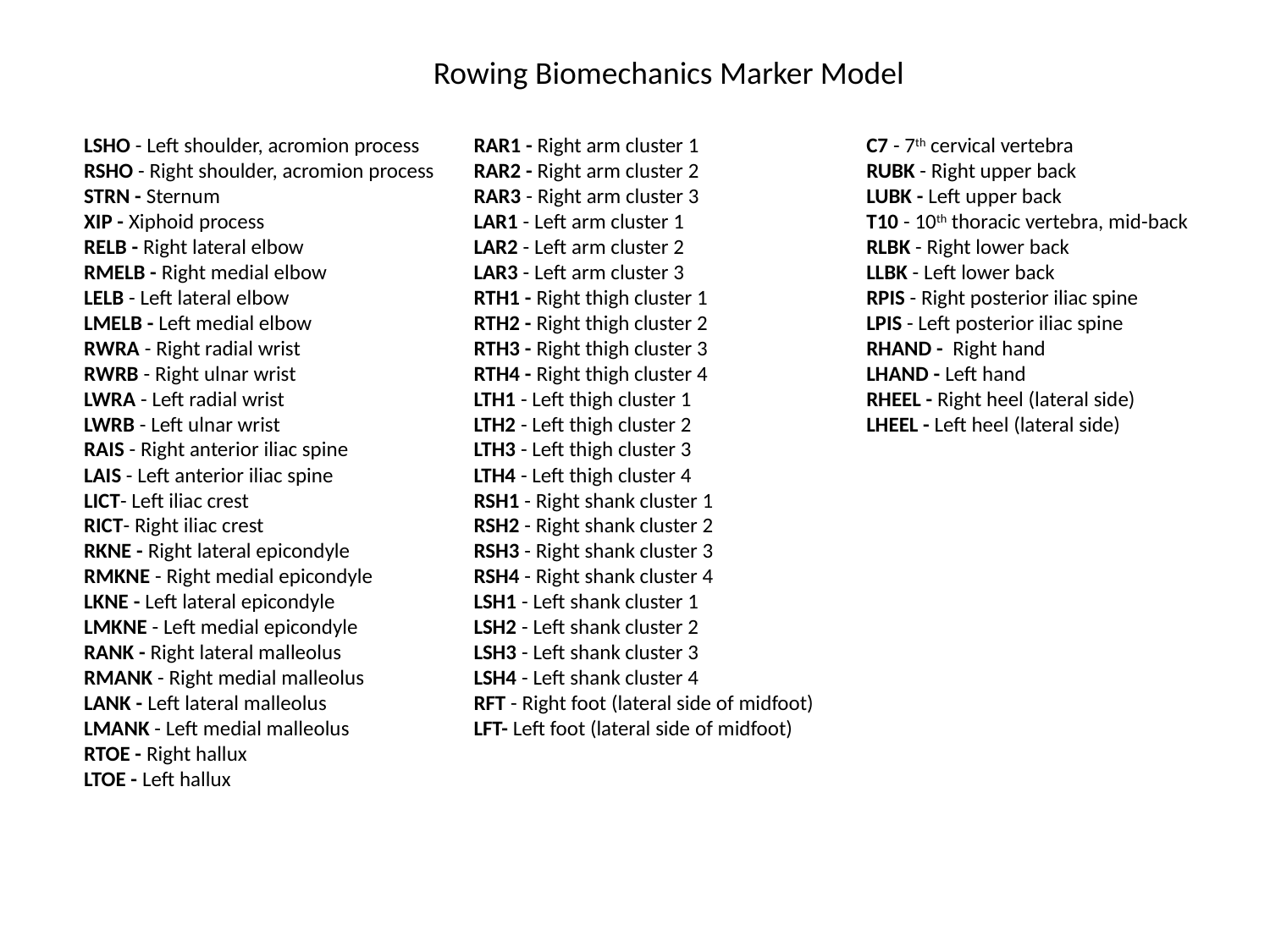

Rowing Biomechanics Marker Model
LSHO - Left shoulder, acromion process
RSHO - Right shoulder, acromion process
STRN - Sternum
XIP - Xiphoid process
RELB - Right lateral elbow
RMELB - Right medial elbow
LELB - Left lateral elbow
LMELB - Left medial elbow
RWRA - Right radial wrist
RWRB - Right ulnar wrist
LWRA - Left radial wrist
LWRB - Left ulnar wrist
RAIS - Right anterior iliac spine
LAIS - Left anterior iliac spine
LICT- Left iliac crest
RICT- Right iliac crest
RKNE - Right lateral epicondyle
RMKNE - Right medial epicondyle
LKNE - Left lateral epicondyle
LMKNE - Left medial epicondyle
RANK - Right lateral malleolus
RMANK - Right medial malleolus
LANK - Left lateral malleolus
LMANK - Left medial malleolus
RTOE - Right hallux
LTOE - Left hallux
RAR1 - Right arm cluster 1
RAR2 - Right arm cluster 2
RAR3 - Right arm cluster 3
LAR1 - Left arm cluster 1
LAR2 - Left arm cluster 2
LAR3 - Left arm cluster 3
RTH1 - Right thigh cluster 1
RTH2 - Right thigh cluster 2
RTH3 - Right thigh cluster 3
RTH4 - Right thigh cluster 4
LTH1 - Left thigh cluster 1
LTH2 - Left thigh cluster 2
LTH3 - Left thigh cluster 3
LTH4 - Left thigh cluster 4
RSH1 - Right shank cluster 1
RSH2 - Right shank cluster 2
RSH3 - Right shank cluster 3
RSH4 - Right shank cluster 4
LSH1 - Left shank cluster 1
LSH2 - Left shank cluster 2
LSH3 - Left shank cluster 3
LSH4 - Left shank cluster 4
RFT - Right foot (lateral side of midfoot)
LFT- Left foot (lateral side of midfoot)
C7 - 7th cervical vertebra
RUBK - Right upper back
LUBK - Left upper back
T10 - 10th thoracic vertebra, mid-back
RLBK - Right lower back
LLBK - Left lower back
RPIS - Right posterior iliac spine
LPIS - Left posterior iliac spine
RHAND - Right hand
LHAND - Left hand
RHEEL - Right heel (lateral side)
LHEEL - Left heel (lateral side)

## Slide 4
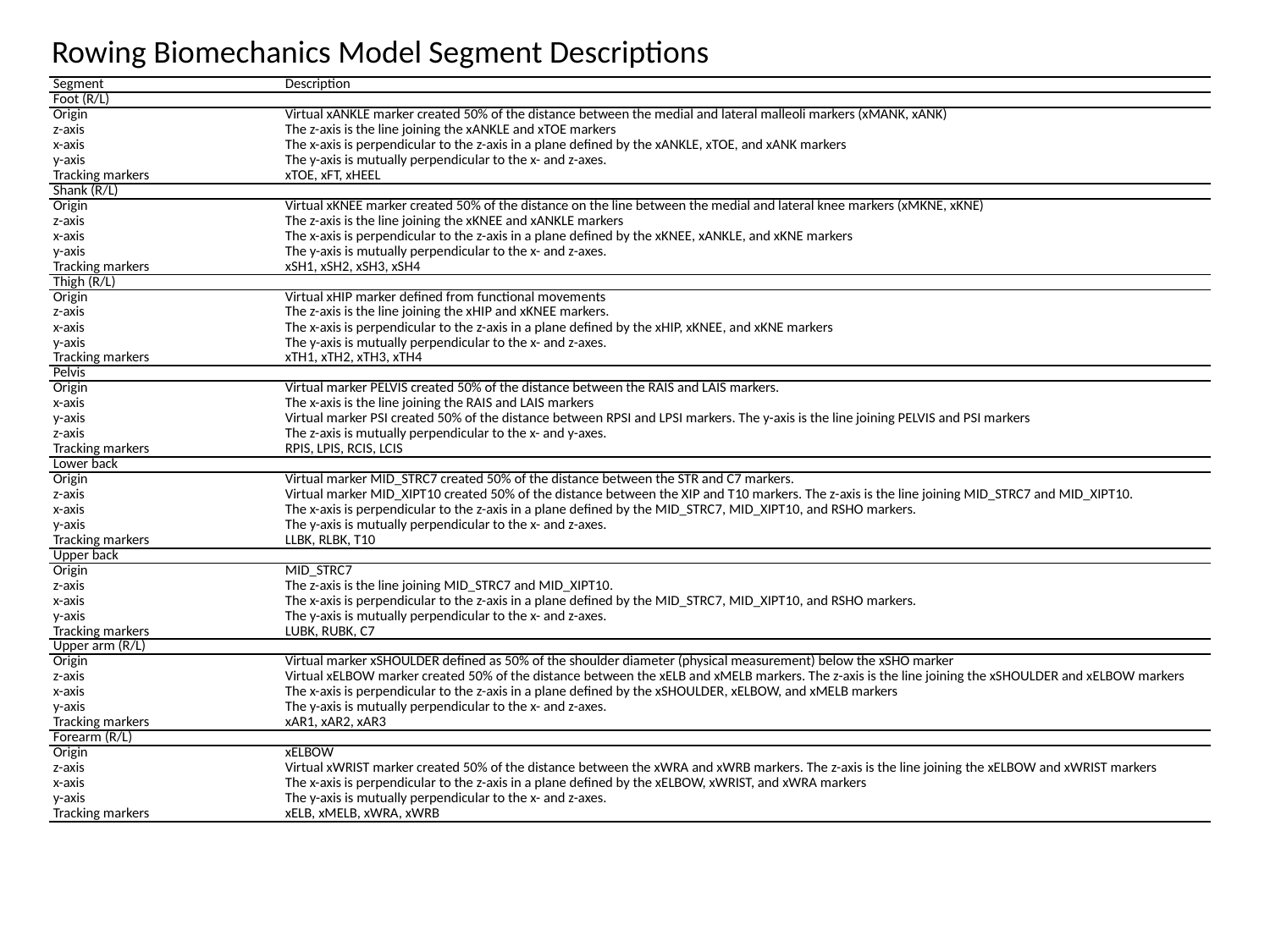

Rowing Biomechanics Model Segment Descriptions
| Segment | Description |
| --- | --- |
| Foot (R/L) | |
| Origin | Virtual xANKLE marker created 50% of the distance between the medial and lateral malleoli markers (xMANK, xANK) |
| z-axis | The z-axis is the line joining the xANKLE and xTOE markers |
| x-axis | The x-axis is perpendicular to the z-axis in a plane defined by the xANKLE, xTOE, and xANK markers |
| y-axis | The y-axis is mutually perpendicular to the x- and z-axes. |
| Tracking markers | xTOE, xFT, xHEEL |
| Shank (R/L) | |
| Origin | Virtual xKNEE marker created 50% of the distance on the line between the medial and lateral knee markers (xMKNE, xKNE) |
| z-axis | The z-axis is the line joining the xKNEE and xANKLE markers |
| x-axis | The x-axis is perpendicular to the z-axis in a plane defined by the xKNEE, xANKLE, and xKNE markers |
| y-axis | The y-axis is mutually perpendicular to the x- and z-axes. |
| Tracking markers | xSH1, xSH2, xSH3, xSH4 |
| Thigh (R/L) | |
| Origin | Virtual xHIP marker defined from functional movements |
| z-axis | The z-axis is the line joining the xHIP and xKNEE markers. |
| x-axis | The x-axis is perpendicular to the z-axis in a plane defined by the xHIP, xKNEE, and xKNE markers |
| y-axis | The y-axis is mutually perpendicular to the x- and z-axes. |
| Tracking markers | xTH1, xTH2, xTH3, xTH4 |
| Pelvis | |
| Origin | Virtual marker PELVIS created 50% of the distance between the RAIS and LAIS markers. |
| x-axis | The x-axis is the line joining the RAIS and LAIS markers |
| y-axis | Virtual marker PSI created 50% of the distance between RPSI and LPSI markers. The y-axis is the line joining PELVIS and PSI markers |
| z-axis | The z-axis is mutually perpendicular to the x- and y-axes. |
| Tracking markers | RPIS, LPIS, RCIS, LCIS |
| Lower back | |
| Origin | Virtual marker MID\_STRC7 created 50% of the distance between the STR and C7 markers. |
| z-axis | Virtual marker MID\_XIPT10 created 50% of the distance between the XIP and T10 markers. The z-axis is the line joining MID\_STRC7 and MID\_XIPT10. |
| x-axis | The x-axis is perpendicular to the z-axis in a plane defined by the MID\_STRC7, MID\_XIPT10, and RSHO markers. |
| y-axis | The y-axis is mutually perpendicular to the x- and z-axes. |
| Tracking markers | LLBK, RLBK, T10 |
| Upper back | |
| Origin | MID\_STRC7 |
| z-axis | The z-axis is the line joining MID\_STRC7 and MID\_XIPT10. |
| x-axis | The x-axis is perpendicular to the z-axis in a plane defined by the MID\_STRC7, MID\_XIPT10, and RSHO markers. |
| y-axis | The y-axis is mutually perpendicular to the x- and z-axes. |
| Tracking markers | LUBK, RUBK, C7 |
| Upper arm (R/L) | |
| Origin | Virtual marker xSHOULDER defined as 50% of the shoulder diameter (physical measurement) below the xSHO marker |
| z-axis | Virtual xELBOW marker created 50% of the distance between the xELB and xMELB markers. The z-axis is the line joining the xSHOULDER and xELBOW markers |
| x-axis | The x-axis is perpendicular to the z-axis in a plane defined by the xSHOULDER, xELBOW, and xMELB markers |
| y-axis | The y-axis is mutually perpendicular to the x- and z-axes. |
| Tracking markers | xAR1, xAR2, xAR3 |
| Forearm (R/L) | |
| Origin | xELBOW |
| z-axis | Virtual xWRIST marker created 50% of the distance between the xWRA and xWRB markers. The z-axis is the line joining the xELBOW and xWRIST markers |
| x-axis | The x-axis is perpendicular to the z-axis in a plane defined by the xELBOW, xWRIST, and xWRA markers |
| y-axis | The y-axis is mutually perpendicular to the x- and z-axes. |
| Tracking markers | xELB, xMELB, xWRA, xWRB |
