# Supplementary figures and images for: The effect of foot-stretcher position and stroke rate on ergometer rowing kinematics
Source: PLoS One. 2023 May 11;18(5):e0285676. doi: 10.1371/journal.pone.0285676 (PMC10174490; doi:10.1371/journal.pone.0285676)

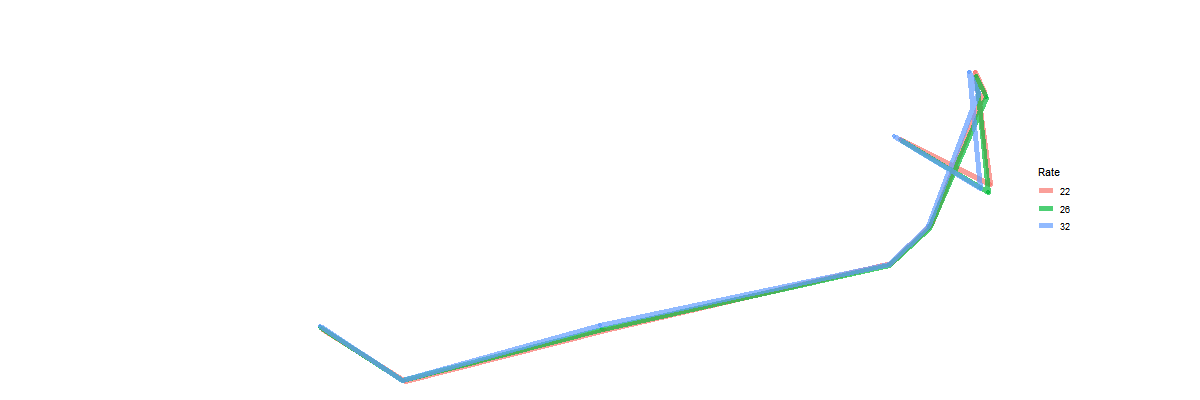

Supplement: S2 File — (GIF) [file pone.0285676.s002.gif]
