# Supplementary material for: The effect of foot-stretcher position and stroke rate on ergometer rowing kinematics
Source: PLoS One. 2023 May 11;18(5):e0285676. doi: 10.1371/journal.pone.0285676 (PMC10174490; doi:10.1371/journal.pone.0285676)
Supplement: Attachment — Submitted filename: Response to Reviewers.docx [file pone.0285676.s003.docx]

*We thank the reviewers for their time and thoughtful reviews. These have led to a much clearer and improved manuscript. Our responses to individual points are detailed below. Changes to the manuscript are highlighted in bold text.*

Reviewer #1: The work is interesting. In my opinion, technologically advanced. However, from the errors, there is a small study group and not heterogeneous. With such a small number of people, I think this may have affected the results.

*Thank you for your review. We acknowledge the concern regarding the heterogeneity of the study group and believe we have addressed these points as described later in this response.*

• L33 - I would suggest adding statistics on how many people in the world practice rowing.

*We agree that this is important context for the study. The most useful information we could find was some survey data from 2017 that estimated the number of rowing machine users in the US to be 11.7 million. We have added this useful information as suggested (L33-35).*

• L37 – ‘’ (Barrett & Manning, 2004).’’ - Incorrect form of quoting

*Thank you, this error has been corrected (L39).*

• Table 1: Participant demographics - Was the number of men and women supported by sample size calculations?

*We have added the following to the methods section:*

*“Given the exploratory nature of the study no formal sample size calculation was performed, however we based our recruitment target on those for similar studies in the prevailing literature [7, 11]” (L92-94)*

*We also included a subgroup analysis to assess if there were any differences in the results for male or female groups, and this did not show any deviations from the main findings. (L195-6)*

Also, why did the authors choose this age group?

*We did not place any age restrictions beyond being over 18 years old. The sample was partially driven by our location at a university with an established rowing program, and by the inclusion criteria needing to find participants who rowed several times per week. It would be interesting to see if there were any differences in the results for other age groups and we have noted this in the limitations (L355-8).*

• L173 – ‘’ Statistical analysis’’ - I suggest adding an effect size and/or confidence interval.
doi: 10.4300/JGME-D-12-00156.1

*As suggested by the reviewer, this table has been added for the timing analysis. (Please note that for the statistical parametric mapping of the kinematic waveforms, the nature of this type of analysis does not easily lend itself to effect size summaries, therefore we have limited these to the discrete variables). (L195)*

• L196 – ‘’ [https://telferbiomech.shinyapps.io/rowingfootposition/](https://urldefense.com/v3/__https:/telferbiomech.shinyapps.io/rowingfootposition/__;!!K-Hz7m0Vt54!ljOscHvy50YOq50kAVVaT7J5_WT3aR_wiAwiKXPgmLd8IzkumNJy9ZLbLmRwBYXAXKP9U3OXfoK-G7s$) ‘’ - this link is not working. You should also add an access date to both links.

*Our apologies, the link has been repaired and access dates added (L206-7)*

• RESULTS - In addition to the graphical form of the presentation of the results, he suggests adding tables with statistical results, this will improve the understanding of the work.

*As suggested by the reviewer, we have presented the results for the timing variables in tabular form. (Please note that for the statistical parametric mapping of the kinematic waveforms, the nature of the analysis does not easily lend itself to being presented in a table form given the volume of points analyzed) (L267)*

• L32 – ‘’ We note a number of limitations’’ - The authors forget to mention the limitations of heterogeneity. Why did the authors choose to study men and women without differentiating this in the later results? There are differences in the biomechanics of men and women. In my opinion, with such a small group of subjects, this may have affected the results.
doi: 10.1038/s41598-020-76674-2
doi: 10.1016/j.gaitpost.2012.04.006
PMID: 26101903

*We have expanded the limitations to comment on this. While there are undoubted differences in biomechanics associated with sex, we do not believe these would affect the overall findings of this repeated measures study, and the results from our sub-group analysis support this. While there are likely absolute differences in kinematics between the males and females in this study, the changes caused by the interventions (stroke rate and foot position) appear to be consistent, therefore we are confident in the generalizability of the findings. (L355-60)*

*“Finally, our sample was made up of young, collegiate age rowers and included both female and male participants. It is possible that other age groups may vary in their response to foot-stretcher position. No differences were found when performing a subgroup analysis by sex and, given the repeated measures design of the study, it is unlikely that this factor would have a significant effect on the overall results.”*

Reviewer #2: The paper presents the study of determining the effects of altering the foot-stretcher position on rowing kinematics across different 20 stroke rates. The data were collected using the Vicon motion capture system. The materials and method part is well written.

*Thank you for the positive feedback*

I suggest to describe the post-processing step in more detail. During motion capturing some markers may disappear. How was the missing trajectory part solved?

We agree this is an important point to expand on. We spent significant time during the project development to optimize our camera set up, as well as the marker model used in order to minimize marker drop-out. Due to the nature of the rowing stroke, some markers were occluded during the experiment, for example the ASIS markers were often completely covered at the catch. In this case additional markers were added to the anterior of the pelvis so that we did not have to rely on the anterior markers for tracking this segment. Using this approach, missing trajectories for tracking marking were minimized (generally under 10 frames) and could be auto-generated using standard splines. (L129-33)

*“The marker model was developed based on preliminary work to minimize marker drop out during the rowing stroke. For example, at the catch, the anterior superior iliac spine markers are generally occluded by the thigh and trunk, therefore additional tracking markers were placed on the posterior of the pelvis to ensure that we could track the segment.”*

Please explain if the participants had a warm-up before rowing?

Yes, please note lines L137-9:

*“After the set up was complete, and prior to the start of the data collection, participants were given at least 10 minutes to warm up using a self-selected protocol on the ergometer with the foot-stretcher in the neutral position.”*
